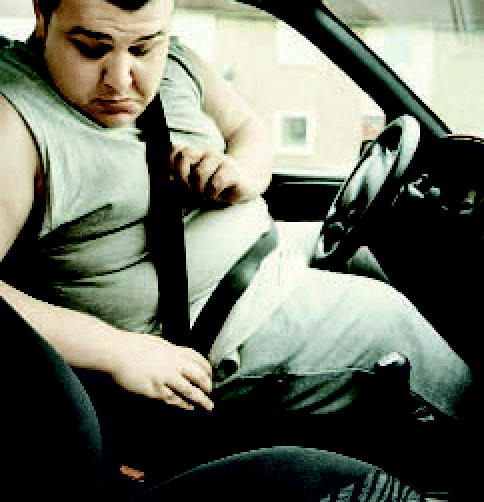# The Beat

**Published:** 2007-01

**Authors:** Erin E. Dooley

## Hazardous Hookahs

Trendy waterpipe cafes have sprung up across the Middle East and are starting to appear in the United States, especially in college towns. According to Georgetown University researcher Christopher Loffredo, patrons of these spots often think the practice of smoking a waterpipe, or hookah, is less harmful than smoking cigarettes. His work has shown that in a typical 30- to 60-minute smoking session, smokers could be inhaling the equivalent of a pack of cigarettes. Loffredo says the practice exposes users to larger amounts of nicotine, carbon monoxide, and other toxicants. Because the tobacco is burned at a lower temperature, smokers find it more tolerable to inhale deeply, and the fact that it takes more pressure to pull air through the waterpipe means tobacco smoke goes deeper into the smoker’s lungs.

**Figure:**
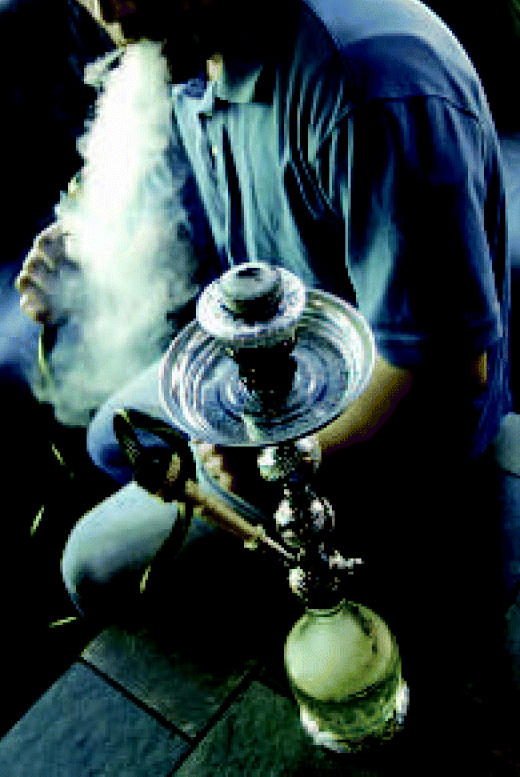


## Asthma Puts the Squeeze on Purses

Asthma-related medical costs in the United States have been estimated at nearly $12.7 billion per year. A new study published in the August 2006 issue of the *Journal of Occupational and Environmental Medicine* has used figures from a large insurance database to get an even more realistic estimate of the costs. The results showed that direct medical costs for persistent asthma sufferers, defined among other criteria as persons experiencing asthma attacks at least twice a week, averaged around $6,500 a year compared to slightly more than $2,000 for patients without asthma. The study also found high indirect costs arose from disability and missed work—more than $900 higher annually for workers with asthma.

## Sewage in Saltwater

The UNEP released its *State of the Marine Environment: Trends and Processes* report in October 2006. This look at oceans and coastal zones states that although a great deal of progress has been made over the past 20 years in reducing oily wastes and industrial chemicals, the marine environment in developing areas is still confronted with the huge problem of sewage. Some 80–90% of sewage entering coastal zones in these areas is raw and untreated. According to the report, this threat, along with the rise in coastal populations and inadequate treatment infrastructure, is jeopardizing not only human health and wildlife but also livelihoods such as fishing and tourism.

**Figure:**
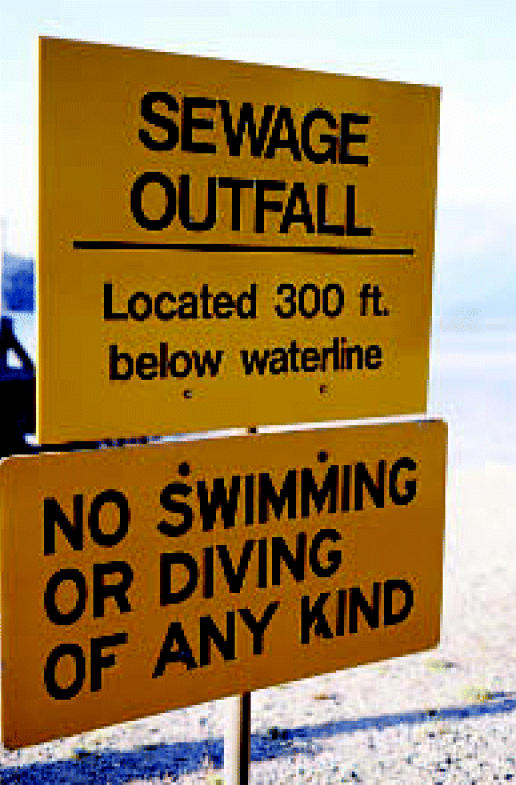


## How Much Water Did *Your* Food Require?

During World Water Week in August 2006, Anders Berntell, head of the Stockholm International Water Institute, suggested that labeling foods to show the amount of water used to produce them could raise awareness among consumers about how much H_2_0 it takes to bring food to market. The UN estimates that it takes a little more than 500 quarts of water to produce a pound of meat, while the same amount of grain takes between 2 and 20 quarts. A report published in August 2006 by the International Water Management Institute states that one-third of the world’s population lives in a region beset by water shortages, and projects that demand for water may double by the year 2050.

**Figure:**
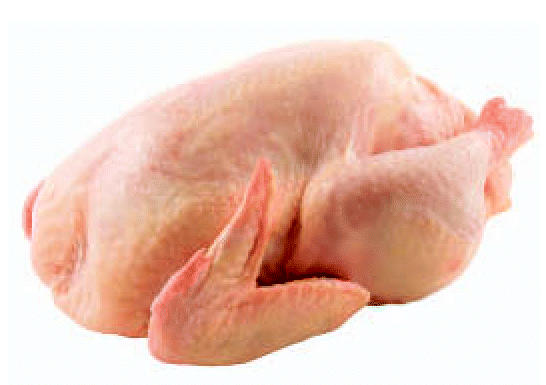


## Copper and Nickel in Chinese Soil

The economic boom in China has increased the consumption of metals by the country’s industrial and manufacturing sectors. A new Australian and Chinese industry-sponsored research effort was launched in Beijing in September 2006 with the aim of developing scientific guidelines for safe levels of copper and nickel in Chinese soils. Earlier studies in Southeast Asia found that soils in the region have low background metal concentrations but ecologically are very sensitive to the addition of metals, as reflected in effects on plant growth and soil microbe function. Under the project, field and laboratory experiments will be conducted on a range of soil and environmental conditions in China to determine the behavior and toxicity of copper and nickel in the soils. The data will be meshed with previously collected data from many other countries to develop toxicity models.

## Too Many Full Tanks?

According to DHHS data, the weight of the average American increased by more than 24 pounds between the years 1960 and 2002. In the October–December 2006 issue of *The Engineering Economist*, scientists from the University of Illinois at Urbana–Champaign and Virginia Commonwealth University calculate that the strain this extra body weight puts on fuel economy means Americans are now pumping 938 million gallons of fuel more per year than they were in 1960—a total of $7.7 million worth each day at $3 per gallon. The authors ruled out factors such as increased cargo weight and decreased fuel efficiency resulting from poor maintenance, and predict these costs will rise further as rates of obesity increase.

**Figure:**